# Development of a Species-Specific PCR Assay for *Aerococcus urinaeequi* Using Whole Genome Sequencing

**DOI:** 10.3390/pathogens14070634

**Published:** 2025-06-25

**Authors:** Hailong Wang, Haixia Li, Zhenxiang Lu, Wenchao Li, Weina Guo

**Affiliations:** 1 College of Animal Science, Anhui Science and Technology University, Fengyang 233100, China; yjs2023443@ahstu.edu.cn (H.W.); yjs2023428@ahstu.edu.cn (H.L.); luzx@ahstu.edu.cn (Z.L.); liwc@ahstu.edu.cn (W.L.); 2 Anhui Province Key Laboratory of Animal Nutritional Regulation and Health, Fengyang 233100, China

**Keywords:** *Aerococcus urinaeequi*, identify, whole genome sequencing and analysis, PCR

## Abstract

*Aerococcus urinaeequi* is an opportunistic pathogen that has been isolated from humans, pigs, and chickens, but with no reports in geese until now. This research aimed to isolate and identify *A. urinaeequi* from four geese, and establish a specific PCR detection method for *A. urinaeequi*. Strain E1 was identified as *A. urnaeequi* through a combination of Gram staining (Gram-positive coccus), colony morphology (α-hemolysis), and whole genome sequencing analysis. Comparative genomics was used to analyze the genome sequences of five reference strains of *A. urinaeequi* to screen for a species-specific genomic region (401 bp). Based on this region, specific primers were designed to establish the PCR detection method for *A. urnaeequi*, and the specificity and sensitivity of this assay were tested. The results showed that the target sequence was specifically amplified only for the genome of *A. urinaeequi*, and that the minimum nucleic acid detection concentration was 7.08 × 10^−3^ ng/μL. The mouse infection model indicated that the target fragment could be amplified from the tissue samples of dead mice in the challenge groups, verifying the applicability of PCR for clinical sample detection. Specific sequences of *A. urinaeequi* were detected in the lungs of three pigs using the PCR method, confirmed to be consistent through whole genome sequencing, and previously identified as *A. urinaeequi* or *A. viridans* by 16S rRNA sequencing. For the detection of fecal samples from geese, canines, and felines using the PCR method, the highest positive rate was 36.9% (31/84) of geese, followed by 21.7% (20/90) of felines, and finally 6.9% (16/230) of canines. A strain of *A. urinaeequi* was isolated and identified in geese for the first time, and a species-specific PCR detection method for *A. urinaeequi* was established with high specificity and sensitivity, which could well distinguish the bacterial species *A. urinaeequi* from its phylogenetically related species, *A. viridans*.

## 1. Introduction

The genus *Aerococcus* is a Gram-positive, microaerophilic, and hydrogen peroxide-negative bacterium [1], which was first proposed by Williams et al. [2]. There are currently 13 species in the genus of *Aerococcus*, including *Aerococcus viridans* (*A. viridans*) [1], *A. urinaeequi* [3], *A. urinae* [4], *A. christensenii*, *A. sanguinicola*, *A. urinaehominis*, *A. suis*, and *A. vaginalis* [5]. Moreover, five new species have been reported recently, including a strain of *A. agrisoli* sp. nov. from a paddy soil sample [6], *A. loyolae* sp. nov., *A. mictus* sp. nov., and *A. tenax* sp. nov., all reclassified and named separately from *A. urinae* [7], and *A. kribbianus* sp. nov., from pig manure [8]. *Aerococcus* is a potential opportunistic pathogen widely found in soil, air, livestock, and the medical environment. *Aerococcus* may cause opportunistic infection in humans [9,10,11,12]. In recent years, *Aerococcus* has been isolated from clinical samples of animals, such as milk produced by cows suffering from mastitis [13], the urine of pigs with a urinary system infection [14], and lobsters suffering from septicemia [15].

*A. urinaeequi* is a Gram-positive, spore-free, non-motile facultative anaerobic coccus in pairs, tetrads, or clusters [3]. The original taxonomic classification of this species was *Pediococcus urinaeequi* (genus *Pediococcus*). In 2005, Felis et al. reclassified it into the genus *Aerococcus* based on DNA–DNA hybridization data and phylogenetic analysis of 16S rRNA gene sequences, renaming it *A. urinaeequi* [3]. So far, *A. urinaeequi* has been isolated from ascites of women with chronic kidney disease [1], pig nasal swabs [16], and laying hens with foot dermatitis [17].

The sequencing of the 16S rRNA gene has been commonly used for species identification, but the method is limited as well. When the 16S rRNA sequences of bacteria are highly homologous, it is not easy to distinguish them [18]. For example, *A. urinaeequi* and *A. viridans* have been reported to exhibit a mere two-base-pair difference in their 16S rRNA gene sequences [3]. DNA–DNA hybridization (DDH) has always been considered the gold standard for identifying bacteria, but because of its cumbersome operation and the difficulty in standardizing among different laboratories, it is usually used for the identification of different strains when the 16S rRNA homology between strains is above 97% [19]. Compared with 16S rRNA sequencing, whole genome sequencing (WGS) provides more comprehensive genetic information and offers higher accuracy in bacterial species identification [20]. However, WGS requires specialized infrastructure and high-throughput sequencing platforms, which are not yet widely accessible in routine laboratories or clinical settings, coupled with their time-consuming workflow.

Current reports on the pathogenicity of *Aerococcus* primarily focus on *A. viridans* and *A*. *urinae* [9,10,11,12,13,14,15], while *A. urinaeequi* has rarely been investigated, and there is a risk of misidentification as *A. viridans* because of the 16S rRNA gene sequences of *A. urinaeequi* and *A. viridans* only differing by 1–2 bases [21]. Therefore, it is necessary to establish a simple, rapid, and accurate PCR-based method for detecting *A. urinaeequi*. To develop such a method, we first needed to obtain reliable genomic data of *A. urinaeequi* as a reference, and this opportunity arose from a natural outbreak of *A. urinaeequi* infection in geese.

In April 2024, four diseased and dead geese from a goose farm (Fengyang County, Chuzhou city, Anhui Province) were subjected to autopsy, and hepatosplenomegaly, turbid and thickened air sacs, and extensive intestinal bleeding were observed. Liver samples of the diseased and dead geese were collected for isolation and the identification of pathogens, as well as whole genome sequencing, average nucleotide identity (ANI) analysis, and non-redundant protein database comparison (NR). The whole genome sequences of the five reference strains of *A. urinaeequi* were compared and analyzed, and specific primers were designed to establish a PCR detection method, which will provide technical support for the clinical detection and identification of *A. urinaeequi*.

## 2. Materials and Methods

### 2.1. Experimental Materials

Blood agar plates were purchased from Changde Beekman Biotechnology Co., Ltd. (Changde, China), BHI medium was purchased from Qingdao Haibo Bio-technology Co., Ltd. (Qingdao, China), the bacterial genomic DNA isolation kit was purchased from Tiangen Biochemical Technology (Beijing) Co., Ltd. (Beijing, China), and the Tissue/Cell/Blood genomic DNA extraction kit was purchased from Wuhan Saiwell Biotechnology Co., Ltd. (Wuhan, China). The strains of *A. viridans* AHFY, *Proteus mirabilis* WHZ2, *Pasteurella multocida* HN-1, *Clostridium perfringens* CQ1, *Enterococcus faecalis* F1, *Salmonella* sms, *Staphylococcus aureus* ptqj, *Bacillus subtilis* KC1, and *Escherichia coli* 97 were isolated and preserved by the laboratory of animal medicine, Anhui Science and Technology University. The reference strain of *A. viridans* ATCC 11563 was purchased from Wuhan Huizao Biotechnology Co., Ltd. (Wuhan, China). Kunming mice were purchased from Hangzhou Ziyuan Experimental Animal Technology Co., Ltd. (Hangzhou, China).

### 2.2. Isolation and Morphological Observation

The liver tissues of the diseased and dead geese were collected and directly streaked on a blood agar medium using a sterile inoculation loop. Single colonies with characteristics of *A. urinaeequi* were picked up and purified on blood agar medium after overnight culture at 35 °C. After purification, single colonies were selected for Gram staining to observe the morphological characteristics of bacteria.

### 2.3. 16S rRNA Amplification and Sequencing Analysis

The DNA of the isolates was extracted as templates using a rapid bacterial genomic DNA isolation kit, and universal primers of bacterial 16S rRNA (upstream 27-F: 5′-AGAGTTTGATCATGGCTCAG-3′ and downstream 1525-R: 5′-AAGGAGGTGATCCAACC-3′) were used for PCR amplification. PCR products were detected via agarose gel electrophoresis, and those with the target band were sent to General Biology (Anhui) Co., Ltd. (Chuzhou, China) for sequencing. The sequencing results of 16S rRNA were blasted in the rRNA/ITS database on NCBI, and the phylogenetic tree was constructed by the software of MEGA11 (version 11.0.13).

### 2.4. Whole Genome Sequencing and Analysis

#### 2.4.1. Whole Genome Sequencing

The purified bacteria were sent to Sangon Biotech (Shanghai) Co., Ltd. (Shanghai, China) for whole genome sequencing. The Illumina NovaSeq v.3.15.4 was used to generate raw second-generation sequencing data, which was then subjected to statistical analysis and quality assessment using Fastp (version 0.23.1) [22]. Quality shearing was performed to obtain processed data with improved accuracy. Subsequently, the second-generation sequencing data was assembled into contigs via SPAdes (version 3.5.0) [23], and GAP within the contigs was supplemented using GapFiller (version 1.11) [24]. Sequence correction was performed using PrInSeS-G (version 1.0.0) to rectify base-calling errors and small insertions/deletions (indels) during the assembly process [25]. Gene elements (CDS, tRNA, and rRNA) were predicted using the NCBI-PGAP (version 1.2.1)/Prokka (version 1.10) software [26]. Finally, a circular whole genome map of strain E1 was generated with Cgview (https://proksee.ca/ (accessed on 19 June 2025)) [27].

#### 2.4.2. Annotation of NR Database

The whole genome protein-coding gene sequences of the isolates were compared in an NR database based on Diamond software (version 2.1.8) [28], and the species information in the annotation results was statistically analyzed.

#### 2.4.3. Analysis of ANI

The whole genome sequences of 22 reference strains of the genus *Aerococcus* (5 strains of *A. urinaeequi*, 3 strains of *A. viridans*, 3 strains of *A. urinae*, 1 strain of *A. sanguinicola*, 1 strain of *A. urinaehominis*, 2 strains of *A. christensenii*, 3 strains of *A. loyolae*, 2 strains of *A. mictus*, and 2 strains of *A. tenax*) were downloaded from the NCBI database (https://www.ncbi.nlm.nih.gov/ (accessed on 14 December 2024)), and the accession numbers of the 22 strains are shown in Table 1. The ANI of strain E1 and the above 22 strains of the genus *Aerococcus* were calculated using the online platform JSpeciesWS (https://jspecies.ribohost.com/jspeciesws/ (accessed on 12 March 2025)) [29]. Then, the ANI values of the 23 strains were obtained, and the ANI data were processed using TBtools-II software (version 2.310) [30] to plot the heat map of the correlation clustering labels.

### 2.5. Establishment of PCR Assay

#### 2.5.1. Screening for Specific Sequence and Designing Primers

The complete genome sequencing maps of five reference strains of *A. urinaeequi* are currently available in the NCBI database, and detailed strain information is shown in Table 2. The whole genome sequences of five *A. urinaeequi* strains were compared and analyzed based on MAFFT 7.0 (https://mafft.cbrc.jp/alignment/server/index.html (accessed on 13 October 2024)) [31], and a species-specific sequence of the genus *A. urinaeequi* was identified. The screened sequence was compared and analyzed using NCBI-BLAST (https://blast.ncbi.nlm.nih.gov/Blast.cgi (accessed on 7 December 2024)) to test the sequence specificity, and the specific primers were designed by SnapGene^®^ software (version 7.0.2) (from Dotmatics; available at snapgene.com) to establish a PCR assay.

#### 2.5.2. Determination of Optimal Annealing Temperature

The annealing temperature in the PCR detection method was optimized, and 9 gradients were set as 52 °C, 53 °C, 54 °C, 55 °C, 56 °C, 57 °C, 58 °C, 59 °C, and 60 °C to find the optimal annealing temperature. Also, lower temperatures from 47 °C to 51 °C were also tested for non-specific amplification.

#### 2.5.3. PCR-Specific Detection

The genomic DNA of A. urinaeequi isolated in this research and other 10 control strains (*A. viridans* ATCC 11563, *A. viridans* AHFY, *Proteus mirabilis* WHZ2, *Pasteurella multocida* HN-1, *Clostridium perfringens* CQ1, *Enterococcus faecalis* F1, *Salmonella* sms, *Staphylococcus aureus* ptqj, *Bacillus subtilis* KC1, and *Escherichia coli* 97) were extracted as templates using a bacterial genomic DNA extraction kit to detect the specificity of the PCR assay.

#### 2.5.4. PCR Sensitivity Test

The bacterial genomic DNA extraction kit was used for extracting the genomic DNA of *A. urinaeequi*, and the nucleic acid concentration was detected to be 70.8 ng/μL via spectrophotometer. The DNA template of *A. urinaeequi* was diluted by a 10-fold gradient (10^−1^~10^−7^) to test the sensitivity of this PCR method.

#### 2.5.5. Artificial Infection of Mice and PCR Detection for *A. urinaeequi*

Twenty-four Kunming mice were divided into 4 groups randomly, and there were six mice in each group, which were housed in separate cages and provided with sufficient water and food. Groups 1, 2, and 3 were set as the challenge groups, while group 4 was the control group. *A. urinaeequi* was cultured in BHI medium for 12 h at 35 °C, and the bacterial suspension was adjusted to three different concentrations, 1.05 × 10^7^, 1.05 × 10^8^, and 1.05 × 10^9^ CFU/mL. The mice in groups 1, 2, and 3 had intraperitoneal injection with a 0.5 mL bacterial suspension of 1.05 × 10^7^, 1.05 × 10^8^, and 1.05 × 10^9^ CFU/mL, respectively. In contrast, the control group received 0.5 mL of BHI medium. The mice were continuously observed for 7 days after the injection, and statistical analysis on the survival rate of the mice in challenge groups was carried out. The organs and tissues of the heart, liver, spleen, lung, kidney, and small intestine were collected from the dead and healthy mice, and the DNA was extracted using the tissue genomic DNA extraction kit for the PCR detection of *A. urinaeequi*.

#### 2.5.6. Detection of *A. urinaeequi* from Clinical Samples by PCR

Lung tissue samples of three pigs were collected for the identification of the pathogen, which was previously identified as *A. urinaeequi* or *A. viridans* via 16S rRNA sequencing, or could not be diagnosed. Therefore, the PCR method established in this research for identification was used, and as well as whole genome sequencing to confirm the results.

A total of 230, 92, and 84 fecal samples were collected from canines, felines, and geese, respectively. Feces were diluted 1000-fold with sterile physiological saline, and then added to LB broth for shaking culture at 37 °C for 12 h. Bacterial DNA was extracted from the culture using a bacterial genomic DNA isolation kit for the subsequent PCR detection of *A. urinaeequi*.

## 3. Results

### 3.1. Colony and Morphology Observation

**Figure 1:**
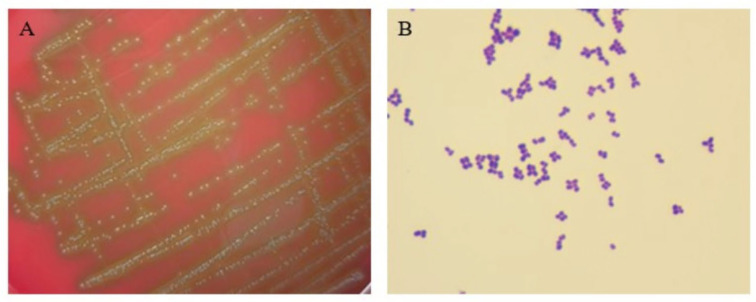
Colony features and morphology observation of the strain. (**A**) Colony characteristics on blood agar medium. (**B**) Morphology observation via Gram staining (1000×).

The isolated bacterium was designated as strain E1, the colony characteristics of which were round, small, gray-white, smooth-surfaced, translucent, and with α-hemolysis on the blood agar medium, as shown in Figure 1A. The Gram staining result of strain E1 was Gram-positive cocci in pairs, tetrads, or clusters, which is shown in Figure 1B.

### 3.2. 16S rRNA Sequencing and Analysis Results

**Figure 2:**
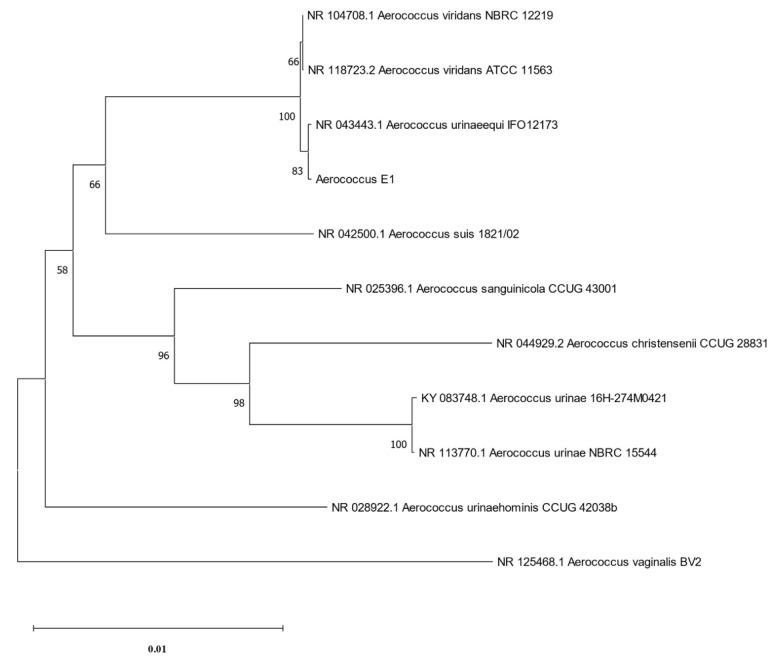
Phylogenetic tree of 16S rRNA. The phylogenetic tree was constructed based on 16S rRNA sequences of strain E1 and 10 strains of *Aerococcus.*

The phylogenetic tree was constructed using the software MEGA11 (version 11.0.13) [32], based on the 16S rRNA of strain E1 and 10 strains of *Aerococcus*, including *A. viridans*, *A. urinaeequi*, *A. suis*, *A. sanguinicola*, *A. christensenii*, *A. urinae*, *A. urinaehominis* and *A. vaginalis*, and it is shown in Figure 2. The results indicated that strain E1 belonged to the same cluster as *A. viridans* and *A. urinaeequi*, and that the similarity of the 16S rRNA of strain E1 to that of strains NBRC 12219 and ATCC 11563 of *A. viridans* and strain IFO12173 of *A. urinaeequi* was 99.65%, 99.24%, and 99.86%, respectively. In view of the sequence similarity of 16S rRNA being above 99% between strain E1 and both *A. urinaeequi* and *A. viridans*, strain E1 could only be identified at the genus level as *Aerococcus*.

### 3.3. WGS Analysis Results

**Figure 3:**
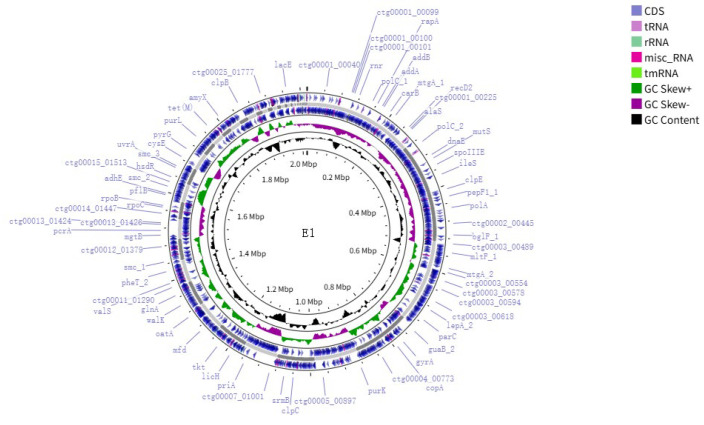
Whole genome circular map of strain E1. The representation from inside to outside is as follows: the first circle is the scale, the second circle is the GC content, the third circle is the GC skew, the fifth circle is the framework of the whole genome sequence, and the fourth and sixth circles are the positions of CDS sequences, tRNAs, rRNAs, misc_RNAs, and tmRNA in the genome.

The whole genome sequence of strain E1 was uploaded to DDBJ/ENA/GenBank, and the registration number was JBLEBE000000000. The whole genome size of E1 was 2 015 846 bp, and the GC content was 39.1%. A genome circle plot was drawn based on the Cgview (https://proksee.ca/ (accessed on 19 June 2025)) [27] online website according to genomic data, which is shown in Figure 3.

#### 3.3.1. NR Annotation Results

**Figure 4:**
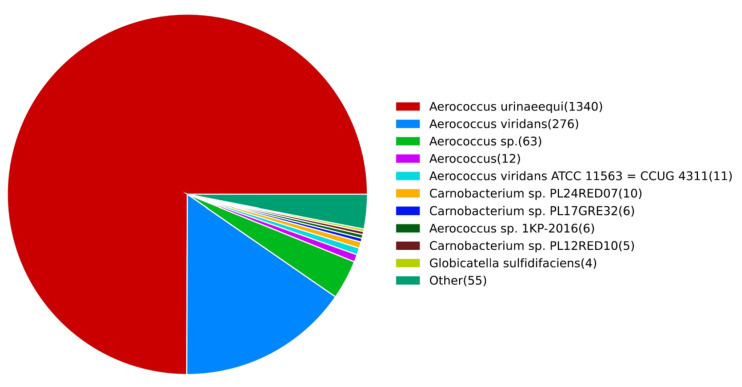
NR annotation results. The number of species-related genes annotated in NR database was listed on the right side, arranged in descending order from high to low.

The results of the NR database comparison are shown in Figure 4, and a total of 1788 genes are annotated. Among them, 1320 genes were annotated in *A. urinaeequi*, and 287 genes in *A. viridans*. The annotation genes of *A. urinaeequi* accounted for the highest proportion in the whole genome of strain E1, which further supports its identification as *A. urinaeequi*.

#### 3.3.2. ANI Analysis Results

**Figure 5:**
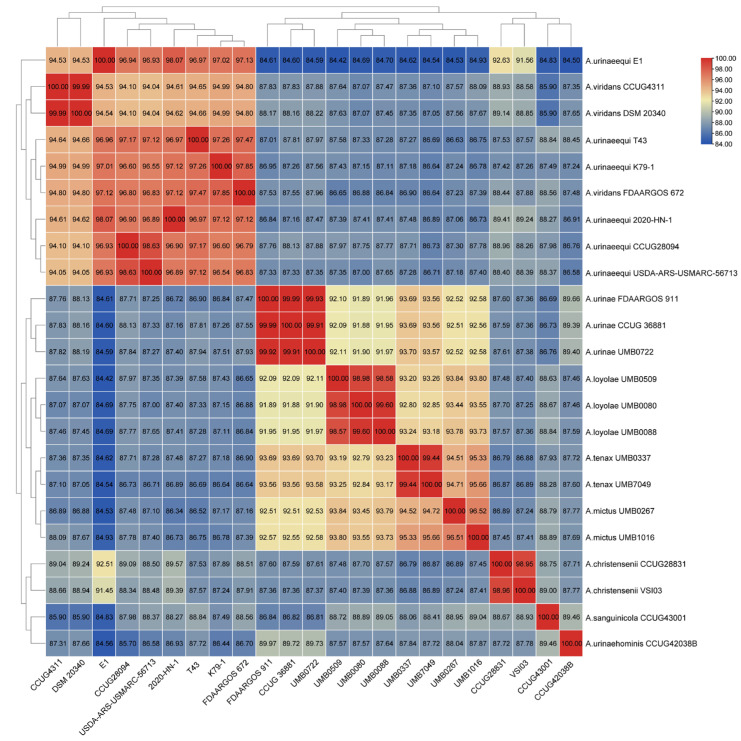
The correlation cluster heat map. The correlation cluster heat map was drawn by the ANI value between strain E1 and 22 strains of *Aerococcus*, which included specific ANI values in each grid; the darker the color of the grid, the higher the value.

The whole genome sequence between strain E1 and 22 other strains of *Aerococcus* was analyzed based on the ANI, and the similarity between strain E1 and 5 strains of *A. urinaeequi* was found to be higher than 95%, such as in the strains T43, K79-1, 020-HN-1, CCUG28094, and USDA-ARS-USMARC-56713, which are shown in Figure 5; the darker the color of the grid, the higher the value.

### 3.4. Establishment of the PCR Method

#### 3.4.1. Specific Sequence Screening Results and PCR Establishment

**Figure 6:**
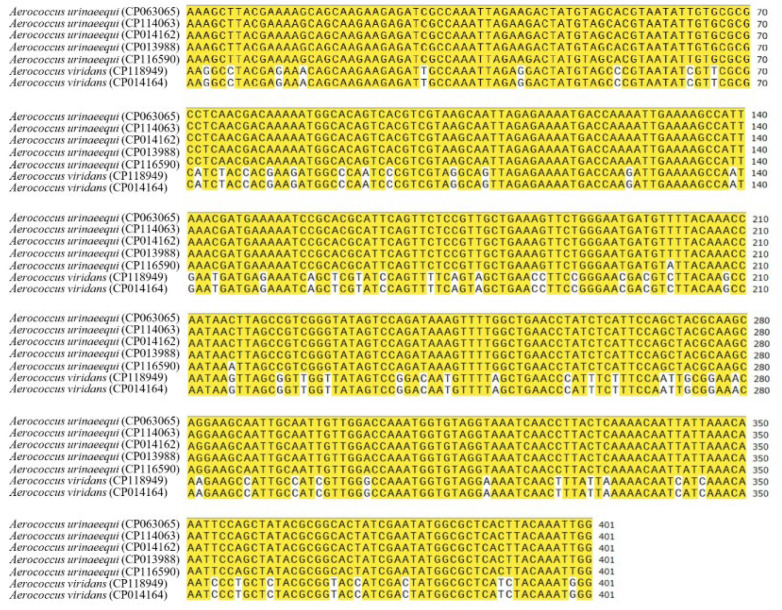
Comparison of the specific sequences. The left side is the strain name and their accession number, and the right side is the specific sequences. The yellow color indicates the same nucleotide in different strains, while the bases without color markings represent differences.

A specific sequence of 401 bp was screened out as the target sequence based on the online software MAFFT 7.0, which was located between the nucleotide 1,852,008 and 1,852,408 of *A. urinaeequi* T43 (GenBank accession number is CP063065, locus tag is IMX20_08495), and belonged to a segment of the gene *abc-f*, which encoded the ABC transporter of type F. A comparison of the specific sequences of 401 bp in 5 strains of *A. urinaeequi* and 2 strains of *A. viridans* is shown in Figure 6. Based on the sequences, a pair of specific primers, upstream primer (AU-F: 5′-AAAGCTTACGAAAAGCAGCA-3′) and downstream primer (AU-R: 5′-CCAATTTGTAAGTGAGCGC-3′), were designed using SnapGene software and synthesized via General Biology (Anhui) Co., Ltd. (Chuzhou, China).

The genomic DNA of *A. urinaeequi* E1 was extracted as a template using a bacterial genomic DNA extraction kit. The PCR reaction system had a total of 50 µL, including 2 μL of DNA template, 2 μL of upstream primer and downstream primer separately, 25 μL of PCR mixture, and 19 μL of sterile water. The annealing temperature was recommended as 54 °C, and the PCR reaction conditions were as follows: pre-denaturation at 95 °C for 5 min, 30 cycles of 95 °C for 30 s, 54 °C for 30 s, and 72 °C for 1 min, and a final extension at 72 °C for 10 min.

#### 3.4.2. Optimization of Annealing Temperature

**Figure 7:**
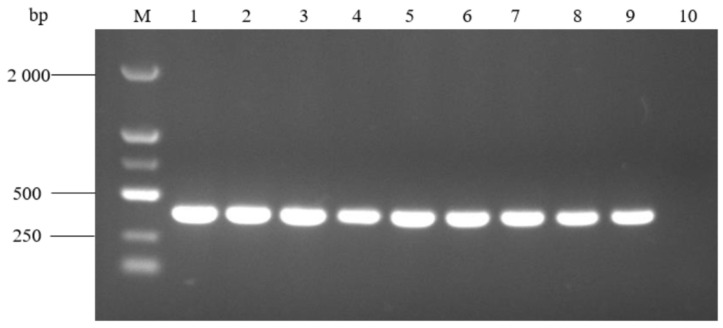
PCR amplification at different annealing temperatures. M: molecular weight marker (2000 bp ladder); lanes 1–9: annealing temperature at 52 °C, 53 °C, 54 °C, 55 °C, 56 °C, 57 °C, 58 °C, 59 °C, and 60 °C, respectively; and lane 10: negative control.

The results of PCR amplification at different annealing temperatures are shown in Figure 7. The target bands were successfully amplified at a temperature of 52 °C~60 °C, and there was no non-specific amplification. Among them, the amplified target band was the brightest at 52 °C, so 52 °C was selected as the optimal annealing temperature. Lower temperatures from 47 °C to 51 °C were also tested for non-specific amplification, and there was no non-specific amplification in any conditions.

#### 3.4.3. Specificity Detection Results

**Figure 8:**
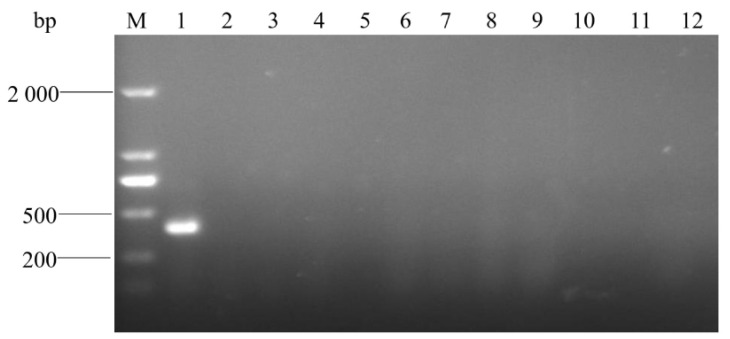
Electrophoresis detection for PCR products. M: molecular weight marker (2000 bp ladder); lanes 1–11: strains of *A. urinaeequi* E1, *A. viridans ATCC* 11563, *A. viridans* AHFY, *Proteus mirabilis* WHZ2, *Pasteurella multocida* HN-1, *Clostridium perfringens* CQ1, *Enterococcus faecalis* F1, *Salmonella* sms, *Staphylococcus aureus* ptqj, *Bacillus subtilis* KC1, and *Escherichia coli* 97, respectively; and lane 12: negative control.

The specificity detection showed that the target band with 401 bp was amplified from the template of *A. urinaeequi* (Figure 8), while the other 9 bacteria species and the negative control were negative and did not amplify the target band. This indicated that the PCR assay established in this research has a high specificity for *A. urinaeequi*.

#### 3.4.4. Sensitivity Test Results

**Figure 9:**
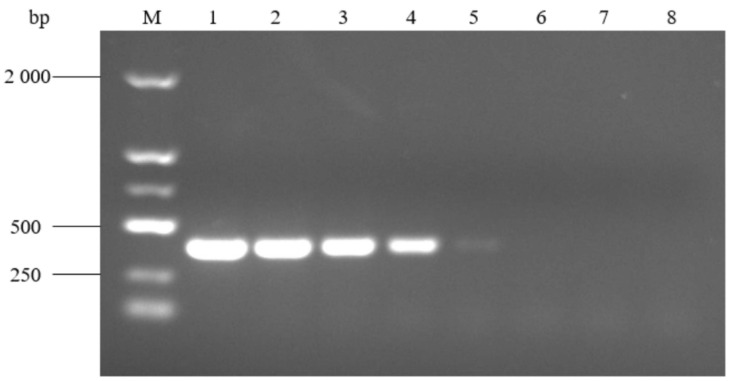
Sensitivity of the PCR detection assay. M: molecular weight marker (2000 bp ladder); lanes 1–7: different dilutions of 10^−1^, 10^−2^, 10^−3^, 10^−4^, 10^−5^, 10^−6^, and 10^−7^, respectively; and lane 8: negative control.

PCR amplification of *A. urinaeequi* E1 with different dilutions of DNA templates (10^−1^~10^−7^) showed that the specific bands were clear and bright at a concentration of 10^−1^~10^−4^ dilution, and that there was also a faint target band at 10^−5^ dilution, so the minimum nucleic acid concentration of this PCR method was 7.08 × 10^−3^ ng/μL (Figure 9).

#### 3.4.5. PCR Detection for *A. urinaeequi* from Artificially Infected Mice

**Figure 10:**
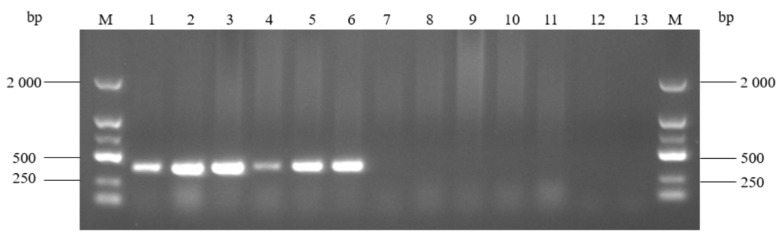
PCR detection of samples from artificially infected mice. M: molecular weight marker (2000 bp ladder); lanes 1–6: heart, liver, spleen, lung, kidney, and small intestine of the dead mice; lanes 7–12: heart, liver, spleen, lung, kidney, and small intestine of the control group mice; and lane 13: negative control.

After artificial infection, mice in group 1 were lethargic and huddled together for 24 h, but all returned to normal thereafter. One mouse died within 24 h in group 2, and two mice died within 24 h and one additional mouse in the next 12 h in group 3. The rest of the mice in the challenge and control groups did not die. In the challenge groups 1, 2, and 3, the survival rates of the mice within 36 h were 100%, 83.33%, and 50%, respectively, while the survival rate of the control group mice was 100%. In autopsy of the dead mice and control group mice, the heart, liver, spleen, lung, kidney, and small intestine were all collected. Then, the DNA from the samples was extracted using the tissue DNA extraction kit and detected via the PCR assay established in this study. As shown in Figure 10, the target bands could be amplified in all samples of the dead mice, but were not detected in the samples of the control group mice. The results indicated that the PCR method could be directly used to detect *A. urinaeequi* from tissue samples of mice, and that there was no need for further isolation or culture.

#### 3.4.6. PCR Detection of *A. urinaeequi* from Clinical Samples

**Figure 11:**
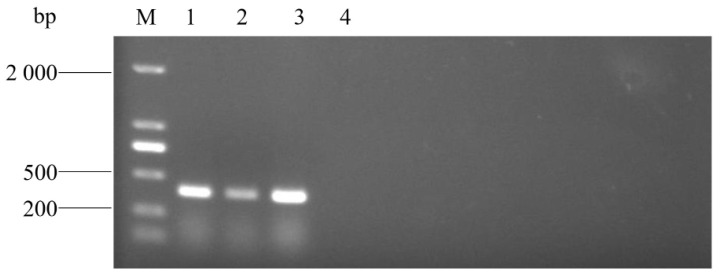
PCR detection of pig samples. M: molecular weight marker (2000 bp ladder); lanes 1–4: AU1, AU2, and AU3; and lanes 4: negative control.

Three strains isolated from lung tissue samples of three dead pigs were further identified via the PCR method established in this study, and specific sequences with 401 bp were all detected (Figure 11). WGS of the three strains identified them as *A. urinaeequi*, consistent with the PCR results, and the relevant sequences were uploaded to the NCBI database with the accession numbers JBPDXK000000000, JBPDXL000000000, and JBPDXM000000000.

Using the PCR method established in this research, *A.urinaeequi* was detected from fecal samples of geese, canines, and felines in different areas, as shown in Table 3. Notably, the highest positive rate was in geese in Anhui province, reaching 36.9% (31/84), followed by felines in Beijing, and finally canines in Beijing and Nantong city of Jiangsu province. For different regions of Anhui, the highest positive rate was 55.6% (5/9) in Dingyuan county, while the lowest positive rate was 30.6% (19/62) in Fengyang county. For canine fecal samples, the positive rate was 8.2% (12/146) in Beijing, whereas in Nantong city, the positivity rate was 4.8% (4/84). For feline fecal samples, the positive rate was 21.7% (20/92) in Beijing. These results indicate that the positive rate of *A.urinaeequi* among different animals in different regions varies to some extent.

## 4. Discussion

In clinical practice, the rapid and accurate detection and diagnosis of bacteria is crucial for disease prevention, control, and treatment. The genus *Aerococcus* is often misidentified as Streptococcus, Enterococcus, or Staphylococcus due to its numerous physiological and biochemical similarities with them [33]. This not only reduces the detection frequency of *Aerococcus*, but also underestimates its potential pathogenicity.

The traditional detection method of *A. urinaeequi* mainly relies on isolation, culture, and biochemical detection. These methods are not only time-consuming, but also prone to interference from surrounding environmental factors, and cannot meet the clinical detection requirements [34]. The identification of *A. urinaeequi* is usually based on biochemical reactions, and commercial biochemical identification systems are widely employed, such as API 20 Strep, Vitek 2 Compact, and BBL-Crystal-GP. However, these systems may lead to misidentification at the species level [35] or insufficient adequate data for the genus *Aerococcus* in their databases. For example, *A. urinaeequi* AV208 was misidentified as *A. viridans* by the API 20 Strep and Vitek 2 Compact systems due to insufficient database coverage [21]. When the API 20 Strep system was used to identify 18 strains of *A. sanguinicola*, there was a misjudgment, as they were incorrectly classified as *A. viridans* [36].

Moreover, it is difficult for 16S rRNA sequencing to effectively distinguish between the strains *A. urinaeequi* and *A. viridans*. For example, the 16S rRNA sequences of strain AV208 of *A. urinaeequi* and strain CCUG4311T of *A. viridans* differed by only one base [21]. Moreover, *A. urinaeequi* IFO 12173 and *A. viridans* ATCC 11563 differed by two bases [3]. In this research, the phylogenetic tree constructed based on the 16S rRNA sequences of 10 strains of the genus *Aerococcus* show that the isolated strain E1 belonged to the same cluster as *A. urinaeequi* and *A. viridans*, so could only be identified as *Aerococcus* spp.

WGS of bacteria has a stronger species classification capability and genomic coverage than 16S rRNA sequencing; it has been applied in most microbiological laboratories and given rise to ANI technology, which is used to achieve an identification function similar to that of DDH [20]. WGS is accurate but time-consuming, costly, and impractical for rapid clinical application. Consequently, developing an alternative diagnostic approach with improved accessibility and efficiency becomes clinically crucial.

The whole genome sequence of E1 consisted of 2 015 846 bp, with a GC content of 39.1%, which was consistent with the GC content of the strains AV208 (39.1%) and 2020-HN-1 (39.04%) of *A. urinaeequi.* The ANI values between E1 and the five strains of *A. urinaeequi* were all higher than 95%, and the highest ANI value was found with the strain 2020-HN-1 (98.07%) of *A. urinaeequi*, while the ANI values of E1 were 94.53% compared with the strains DSM 20340 and CCUG4311 of *A. viridans* [37]. The whole genome sequence of E1 was annotated with a total of 1788 genes in the NR database, among which the highest number of genes (1340) were related to *A. urinaeequi*. In summary, the isolate E1 was identified as *A. urinaeequi*. In addition, the ANI values between the strain FDAARGOS_672 of A. viridans and strains DSM 20340 and CCUG4311 of *A. viridans* were both 94.80%. In comparison, the ANI values between the strain FDAARGOS_672 and strains E1 and T43 of *A. urinaeequi* were 97.13% and 97.47%, respectively, which is shown in Figure 5, indicating that the strain FDAARGOS_672 may be reclassified as *A. urinaeequi* rather than *A. viridans*.

Based on comparative genomics, a specific region of 401 bp of *A. urinaeequi* was screened out, and the specific PCR detection method of *A. urinaeequi* was established. The specific detection of the PCR assay showed that only strain E1 of *A. urinaeequi* was successfully amplified to produce the corresponding target band, and that none of the other species, including the strain AHFY of *A. viridans*, were amplified to the target band; therefore, the PCR method could meet the clinical detection requirements for *A. urinaeequi*. Rubel et al. developed a conventional PCR technique for the detection of *Xanthomonas campestris* pv. *raphanin* that could detect the pathogen directly from artificially infected samples with a minimum nucleic acid detection limit of 7.0 × 10^−4^ ng/μL [38]. Alvandi et al. designed a pair of specific PCR primers and established a PCR detection method for *Xanthomonas translucens* pv. *undulosa* via comparative genomics analysis, with a minimum detection limit of 4.5 × 10^−3^ ng/μL [39]. In this research, the PCR detection assay established for *A. urinaeequi* could be directly detected from the tissues of artificially infected dead mice and the lung tissues of pigs, as well as fecal samples from geese, canines, and felines, and the minimum detection concentration of nucleic acid was 7.08 × 10^−3^ ng/μL. Compared with the above assays, the sensitivity was lower than that of Rubel, and higher than that of Alvandi. Compared with the high cost, long duration, and complex data analysis of WGS, the PCR method for *A. urinaeequi* established in this research has high specificity and sensitivity, low cost, and rapid detection, which can be completed within 3 h from the DNA extraction of tissue samples to the observation of results using a gel imaging system, and the detection efficiency has been greatly improved.

## 5. Conclusions

In this study, a strain of *A. urinaeequi* was isolated and identified from four dead geese through methods such as isolation and culture, microscopic examination, 16S rRNA sequencing, and whole genome sequencing and analysis. Based on comparative genomics analysis, a specific sequence of *A. urinaeequi* was screened out. A pair of specific primers were designed, and a PCR detection method was established. The specificity and sensitivity detection results of this PCR method showed that it had strong specificity and high sensitivity, and could complete the detection within 3 h and effectively distinguish between the bacterial species *A. urinaeequi* and *A. viridans*. Moreover, the PCR method was effective in clinical samples from different animals such as pigs, geese, canines, and felines.

## Figures and Tables

**Table 1:** Accession numbers of *Aerococcus* strains for ANI analysis.

Reference Strain	Accession Number	Reference Strain	Accession Number
*A. christensenii* CCUG28831	CP014159	*A. christensenii* VSI03	CP118095
*A. loyolae* UMB0080	CP126958	*A. loyolae* UMB0088	CP145131
*A. loyolae* UMB0509	CP127213	*A. mictus* UMB0267	CP147497
*A. mictus* UMB1016	CP145132	*A. sanguinicola* CCUG43001	CP014160
*A. tenax* UMB0337	CP142608	*A. tenax* UMB7049	CP145134
*A. urinae* NBRC 15544	BCQH00000000	*A. urinae* FDAARGOS 911	CP065662
*A. urinae* UMB0722	CP145136	*A. urinaeequi* CCUG28094	CP014162
*A. urinaeequi* 2020-HN-1	CP114063	*A. urinaeequi* K79-1	CP116590
*A. urinaeequi* T43	CP063065	*A. urinaequi* CCUG42038B	CP014163
*A. viridans* CCUG4311	CP014164	*A. viridans* DSM 20340	CP118949
*A. viridans* FDAARGOS 672	CP046334	*A. urinaeequi* USDA-ARS-USMARC-56713	CP013988

**Table 2:** Strain information on *A. urinaeequi* for whole genome sequence analysis.

Strain	Host	Country	Year	Accession Number	Genome Size (bp)
CCUG28094	Equus caballus	Denmark	2016	CP014162	2,013,339
USDA-ARS-USMARC-56713	Bos taurus	America	2016	CP013988	2,054,328
2020-HN-1	Duck	China	2022	CP114063	2,133,811
T43	Pig	China	2020	CP063065	2,055,141
K79-1	Bos taurus	Switzerland	2023	CP116590	2,083,683

**Table 3:** Positive rates of *A.urinaeequi* in fecal samples.

Animal Species	Sampling Location	Number of Samples	Positive Rate (Positive/Total)	Total Positive Rate (Positive/Total)
*Geese*	Fengyang, Anhui	62	30.6% (19/62)	36.9% (31/84)
	Dingyuan, Anhui	9	55.6% (5/9)	
	Huaiyuan, Anhui	13	53.8% (7/13)	
*Canines*	Beijing	146	8.22% (12/146)	6.96% (16/230)
	Nantong, Jiangsu	84	4.8% (4/84)	
*Felines*	Beijing	92	21.7% (20/92)	21.7% (20/92)

## Data Availability

The original contributions presented in this study are included in the article; further inquiries can be directed to the corresponding author.
